# DNA methylation-mediated ROS production contributes to seed abortion in litchi

**DOI:** 10.1186/s43897-024-00089-0

**Published:** 2024-04-02

**Authors:** Hanhan Xie, Yedan Zheng, Mengyue Xue, Yulian Huang, Dawei Qian, Minglei Zhao, Jianguo Li

**Affiliations:** State Key Laboratory for Conservation and Utilization of Subtropical Agro-Bioresources, Guangdong Laboratory for Lingnan Modern Agriculture, Key Laboratory of Biology and Genetic Improvement of Horticultural Crops (South China), Ministry of Agriculture and Rural Affairs, Guangdong Litchi Engineering Research Center, College of Horticulture, South China Agricultural University, Guangzhou, 510642 China

**Keywords:** Seed abortion, DNA methylation, ROS

## Abstract

Although there is increasing evidence suggesting that DNA methylation regulates seed development, the underlying mechanisms remain poorly understood. Therefore, we aimed to shed light on this by conducting whole-genome bisulfite sequencing using seeds from the large-seeded cultivar 'HZ' and the abortive-seeded cultivar 'NMC'. Our analysis revealed that the 'HZ' seeds exhibited a hypermethylation level compared to the 'NMC' seeds. Furthermore, we found that the genes associated with differentially methylated regions (DMRs) and differentially expressed genes (DEGs) were mainly enriched in the reactive oxygen species (ROS) metabolic pathway. To investigate this further, we conducted nitroblue tetrazolium (NBT) and 2,7-Dichlorodihydrofluorescein (DCF) staining, which demonstrated a significantly higher amount of ROS in the 'NMC' seeds compared to the 'HZ' seeds. Moreover, we identified that the gene *LcGPX6*, involved in ROS scavenging, exhibited hypermethylation levels and parallelly lower expression levels in 'NMC' seeds compared to 'HZ' seeds. Interestingly, the ectopic expression of *LcGPX6* in *Arabidopsis* enhanced ROS scavenging and resulted in lower seed production. Together, we suggest that DNA methylation-mediated ROS production plays a significant role in seed development in litchi, during which hypermethylation levels of *LcGPX6* might repress its expression, resulting in the accumulation of excessive ROS and ultimately leading to seed abortion.

**Supplementary Information:**

The online version contains supplementary material available at 10.1186/s43897-024-00089-0.

## Core

Our analysis found that the large-seeded cultivar 'HZ' seeds had a higher DNA methylation level than the abortive-seeded cultivar 'NMC' seeds. 'NMC' seeds had significantly more ROS than 'HZ' seeds, and the gene LcGPX6, involved in ROS scavenging, had higher DNA methylation and lower expression than that in 'HZ' seeds, suggesting that DNA methylation-mediated ROS production plays a role in seed development, with higher DNA methylation levels of *LcGPX6* suppressing its expression and leading to excessive ROS accumulation and seed abortion.

## Gene and accession numbers

Sequence data from this article can be found in the litchi genome database (10.1101/2022.11.25.517904) under the accession numbers: *LcGPX6*: LITCHI022143, *LcGPX1/4/7*: LITCHI016878, *LcGPX2*: LITCHI015847, *LcGPX3*: LITCHI015847, *LcGPX5*: LITCHI026281, *LcGPX8*:LITCHI022145, *LcCAT*: LITCHI009991, *LcAPX1*: LITCHI024768, *LcAPX2*: LITCHI018535, *LcAPX3*: LITCHI002021, *LcAPX4*: LITCHI010566, *LcAPX5*: LITCHI006437, *LcAPX6*: LITCHI016989, *LctAPX/Sapx*: LITCHI011263, LcCSD1: LITCHI003782, *LcCSD2*: LITCHI024579, *LcCSD3*: LITCHI003354, *LcFSD1*: LITCHI020212, *LcFSD3*: LITCHI006009, *LcMSD1*: LITCHI027490, *LcPrxR A/B*: LITCHI018945, *Lc*PrxR F: LITCHI014732, *LcPrxR Q*: LITCHI017484, *LcType 2-PrxR B/C/D*: LITCHI016784, *LcType 2-PrxR E*: LITCHI018774, *Lc*RbohA/C: LITCHI020220, *Lc*RbohB: LITCHI030320, *Lc*RbohD: LITCHI021682, *Lc*RbohE: LITCHI017482, *Lc*RbohF/I: LITCHI022022, *Lc*RbohH/J: LITCHI024802, *Lc*RbohG: LITCHI030338.

## Introduction

Seeds serve as the primary source of nutrients for both humans and animals, while also playing a crucial role in ensuring offspring. Therefore, comprehending the intricate mechanisms underlying seed development holds paramount importance in enhancing agricultural practices and effectively managing genetic resources. In the majority of angiosperms, seeds are formed through the process of double fertilization, which culminates in the production of a mature seed comprising the embryo, endosperm, and seed coat.

Recent studies have shed light on the crucial role of epigenetic modifications in seed development. One such modification is DNA methylation, which can modulate chromatin structure and function, thereby influencing the silencing of transposable elements (TEs) and gene expression (Buitrago et al. 2021). In plants, DNA methylation occurs in different sequence contexts, including CG, CHG, and CHH (where H represents A, C, or T). The maintenance of CG methylation is regulated by ETHYLTRANSFERASE 1 (MET1), while CHG methylation is maintained by CHROMOMETHYLASE 2 (CMT2) and CHROMOMETHYLASE 3 (CMT3). Furthermore, CHH methylation is maintained by either CMT2 or DOMAINS REARRANGED METHYLTRANSFERASE 2 (DRM2) (Gallego-Bartolome 2020). Perturbations in DNA methylation, such as a global loss of CG methylation in *Arabidopsis*, have been associated with abnormal embryo development and impaired megaspore mother cell development (FitzGerald et al. 2008; Li et al. 2017). Additionally, disruptions in non-CG methylation in maize have been shown to cause severe defects in ovule development (Garcia-Aguilar et al. 2010). Similarly, reproductive defects have been observed in RdDM mutants in tomato and *Brassica rapa*, with CHH methylation playing a role in chickpea seed development (Gouil and Baulcombe 2016; Grover et al. 2018; Rajkumar et al. 2020). However, loss of non-CG methylation has no effect on seed development in *Arabidopsis* and soybean (Lin et al. 2017). Although it has been demonstrated that DNA methylation plays critical roles in seed development, the precise mechanisms through which it regulates this process remain poorly understood.

Reactive oxygen species (ROS) have been identified as both toxic byproducts of aerobic metabolism and crucial regulators of development, including seed development, in plants. ROS can be generated through various enzymatic activities, with NADPH oxidases (Rboh) being extensively studied in this context. To maintain ROS homeostasis, plants possess ROS-scavenging enzymes including superoxide dismutase (SOD), ascorbate peroxidase (APX), catalase (CAT), glutathione peroxidase (GPX), and peroxiredoxin (PrxR) (Apel and Hirt 2004; Mittler et al. 2004). In *Arabidopsis*, it has been demonstrated that maintaining ROS homeostasis during female gametophyte development is crucial for proper embryo sac patterning and fertilization. ROS are detected in the nucellus during megasporogenesis and the central cell of the embryo sac during megagametogenesis. Pollination leads to an oxidative burst, after which ROS are cleared from the embryo sac (Martin et al. 2013). MnSOD (MSD) has been identified as a pivotal protein that regulates ROS levels during female gametogenesis. Mutations in *MSD*, such as the *oiwa* mutant, disrupt ROS homeostasis, resulting in high ROS levels in the embryo sac and leading to sterility or arrested embryogenesis (Victoria Martin et al. 2013). Recently, there have been reports highlighting the crucial roles of DNA methylation-mediated ROS homeostasis in various aspects of plant development and stress response, including salt stress (Chen et al. 2015; Hu et al. 2021), heat stress(Ma et al. 2018; Sakai et al. 2022; Zhu et al. 2021), chilling/freezing stress(Guo et al. 2019; Zheng et al. 2022), and fruit ripening (He et al. 2020). For instance, the application of 5-azaC, an inhibitor of DNA methyltransferase, has been found to enhance the rates of superoxide anion (O_2_^−^) production, thereby contributing to the ripening of berry fruits (Guo et al. 2019). In the case of rice, it has been speculated that high temperatures induce hypomethylation, which releases genes responsible for ROS production from silencing. This, in turn, leads to an increased generation of H_2_O_2_ and microspore sterility (He et al. 2020). However, whether DNA methylation-mediated ROS homeostasis plays a role in seed development remains unknown.

Litchi (*Litchi chinensis* Sonn.) is an important tropical and subtropical fruit originating in Southern China and is now cultivated in over 20 countries (Hu et al. 2022). The size of the litchi seed is a major factor influencing its economic value. Fruits with smaller or aborted seeds have a higher edible rate, better taste, and greater economic value. In litchi, embryonic development follows three patterns: normal, abortive, and partial abortive. Previous studies have indicated that cultivars with small (abortive)-seeded or partially abortive-seeded characteristics exhibit varying percentages of small (abortive) seeds each year, suggesting that epigenetic factors may regulate litchi seed development (Chu et al. 2015; Xie et al. 2019). However, the precise role of DNA methylation in regulating litchi seed development remains unclear. Therefore, the objective of this study is to investigate whether there are genome-wide differences in DNA methylation between 'HZ' (large-seeded cultivar) and 'NMC' (abortive-seeded cultivar) and identify the key pathways mediated by DNA methylation in regulating litchi seed development. We performed DNA methylation profiling at single-base resolution in the C, CG, CHG, and CHH sequence contexts during early seed development in ‘HZ’ and ‘NMC’. The results revealed higher DNA methylation levels in ‘HZ’ compared to ‘NMC’. By integrating RNA-seq and DNA methylome datasets, we identified key DNA methylation-regulated pathways and genes associated with seed development. Furthermore, we described the function of LcGPX6 in seed development via scavenging ROS.

## Results

### Seed development in the large-seeded cultivar 'HZ' and the abortive-seeded cultivar 'NMC'

**Fig. 1:**
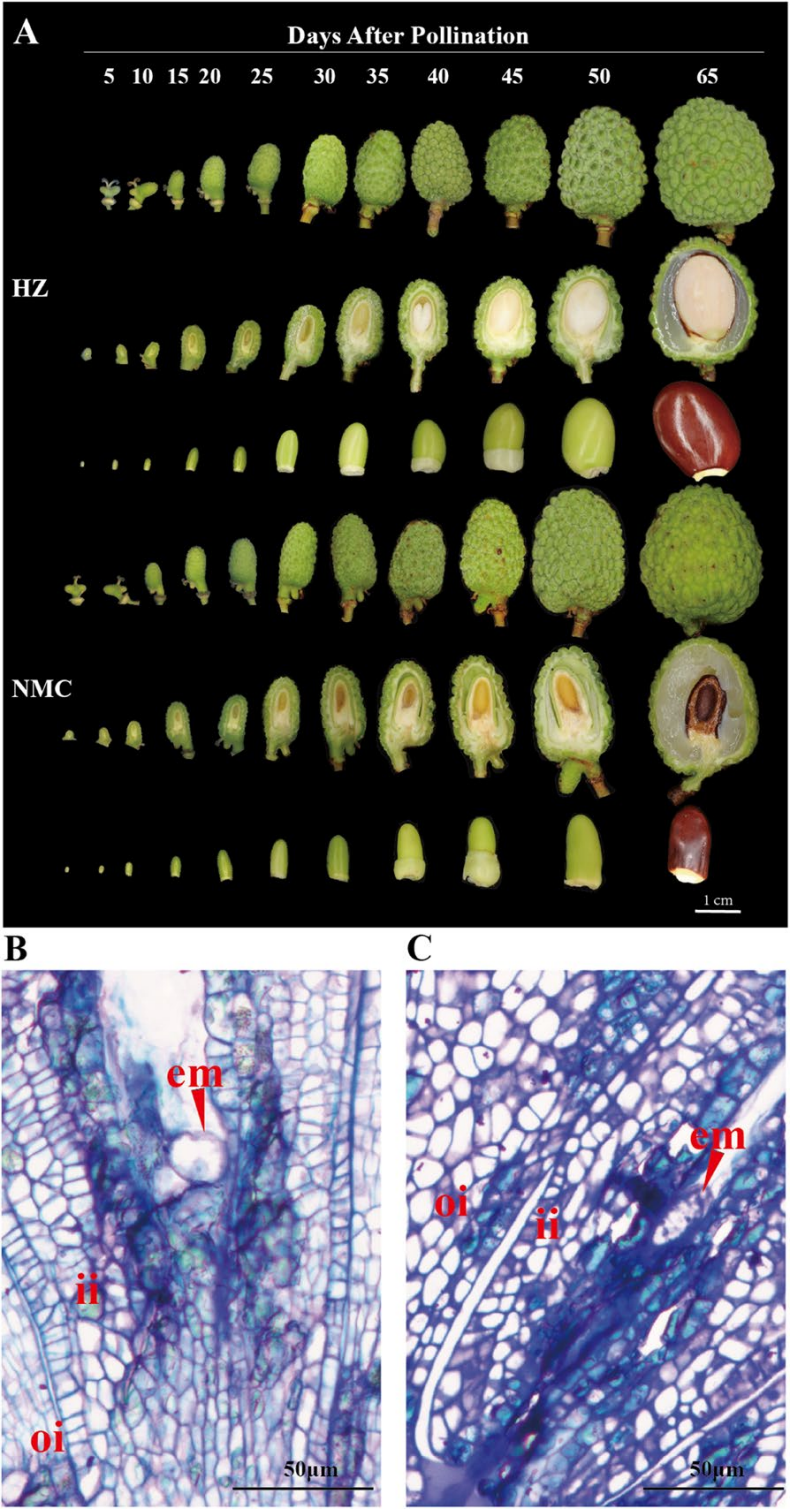
Comparison of seed development between the large-seeded cultivar 'HZ' and the abortive-seeded cultivar 'NMC’. **A** Photos showing the different stages of seed development after pollination (DAP) in ‘HZ’ and ‘NMC’. Bar = 1 cm; The cross-sections of seed at 5 DAP in ‘HZ’ (**B**) and ‘NMC’ (**C**). The red arrows indicate the embryos. em, embryo; ii, inner integument; oi, outer integument. Scale bars = 50 μm

Seed development in the cultivar 'HZ' is deemed normal, resulting in the production of large seeds, while the cultivar 'NMC' exhibits a tendency for early embryo abortion, yielding small abortive seeds (Fig. 1A). To investigate the possibility of disintegration and abnormal division of the zygote in 'NMC', cross-sections of ovules were examined using light microscopy. Furthermore, the development of embryos in 'HZ' and 'NMC' was compared at 5 days after pollination (DAP). The findings revealed that the zygote in 'HZ' remained intact at 5 DAP (Fig. 1B), whereas the zygote in 'NMC' underwent disintegration and abortion (Fig. 1C), indicating that seed abortion in 'NMC' occurs at an early stage.

### DNA methylome characteristics of 'HZ' and 'NMC' seeds at an early developmental stage

To investigate the differences in DNA methylation patterns during zygote formation and division in the cultivars 'HZ' and 'NMC', whole-genome bisulfite sequencing (WGBS) was conducted at single base resolution on 5, 10, and 15 days after pollination (DAP) seeds from both cultivars (Fig. 1).

**Fig. 2:**
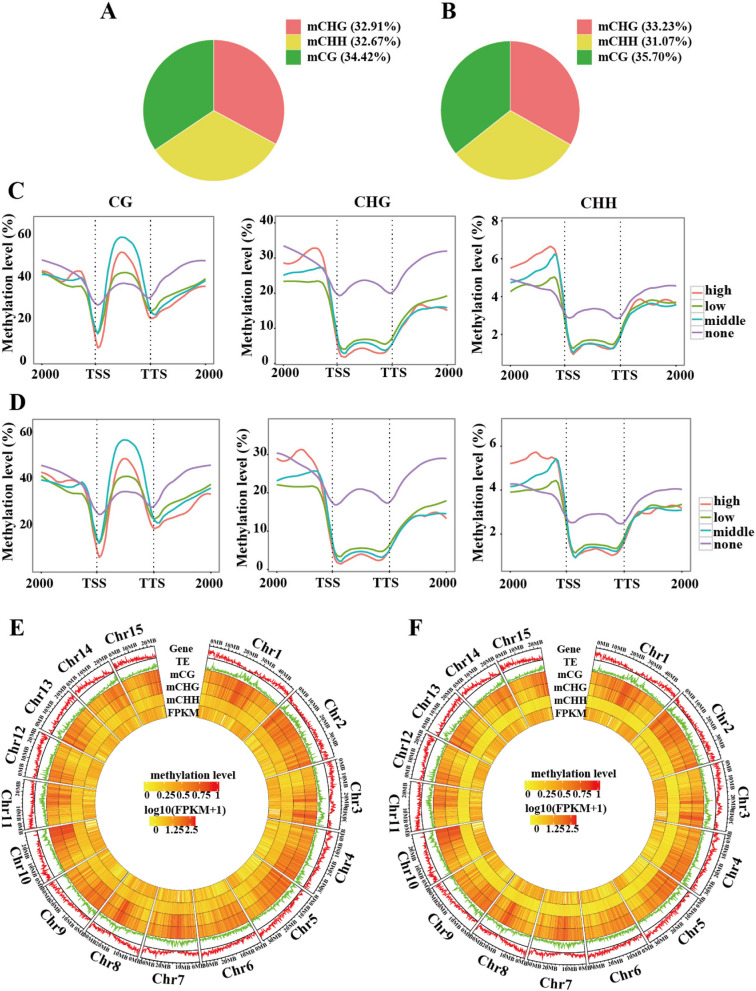
Genome-wide DNA methylation profiling during the early stages of seed development in ‘HZ’ and ‘NMC’. A pie chart indicating the the proportion of mCG, mCHG and mCHH in all methylcytosines (mCs) at 5 DAP in 'HZ' (**A**) and 'NMC' (**B**). The DNA methylation levels within the gene body and ± 2 kb regions of genes are shown for different sequence contexts in gene sets that are expressed at different levels at 5 DAP in 'HZ' (**C**) and 'NMC' (**D**). The expression levels are categorized as high (FPKM > 100), middle (10 < FPKM ≤ 100), low (1 < FPKM ≤ 10), and none (FPKM ≤ 1). The terms TSS and TTS represent the transcription starting site and transcription terminal site, respectively. A chromosome heat map illustrating gene density, transposable elements density, DNA methylation levels in different sequence contexts, and gene expression levels of seeds at 5 DAP in 'HZ' (**E**) and 'NMC' (**F**). The bin size used for this visualization is 100 kb. The outer ring displays the chromosome names and scale

First, we examined the number and proportion of mCG, mCHG, and mCHH sites in 'HZ' and 'NMC' at 5, 10, and 15 DAP (Fig. 2A-B, Table S1). Our findings revealed that, for instance, the proportions of mCG, mCHG, and mCHH sites among all mC sites were 34.42%, 32.91%, and 32.67% in 'HZ' at 5 DAP seeds, and 35.70%, 33.23%, and 31.07% in 'NMC' (Fig. 2A, B), indicating that the proportions of mCG, mCHG, and mCHH sites among all mC sites were similar between 'HZ' and 'NMC'.

To ascertain whether the relationship between DNA methylation and gene transcription in 'HZ' and 'NMC' exhibited similarities, transcriptome sequencing was conducted at 5, 10, and 15 DAP. Genes were categorized into four groups based on their expression levels: high expression level (FPKM > 100), medium expression level (10 < FPKM ≤ 100), low expression level (1 < FPKM ≤ 10), and no expression level (FPKM ≤ 1). In both 'HZ' and 'NMC', we observed a negative correlation between gene expression level and DNA methylation levels at the transcription starting site (TSS) and transcription terminal site (TTS) of genes, downstream regions in CG contexts, and the gene body in CHG and CHH contexts (Fig. 2C-D, S1). However, we noted that genes expressed at high and medium levels displayed higher methylation levels within the gene body in the CG context (Fig. 2C-D, S1). Similarly, DNA methylation levels were lower at the TSS or TTS compared to other regions (Fig. 2C-D, S1). Furthermore, we observed an inverse relationship between gene density and transposable element (TE) density across the 15 chromosomes, with higher TE density regions exhibiting higher DNA methylation levels in both 'HZ' and 'NMC' (Fig. 2E-F, S2). These results suggest that the pattern of DNA methylation-mediated gene transcription and the association between DNA methylation distribution and gene/transposable element (TE) density in 'HZ' are consistent with those observed in 'NMC'.

### DNA methylation levels in ‘HZ’ were higher than ‘NMC’ seeds at an early developmental stage

**Fig. 3:**
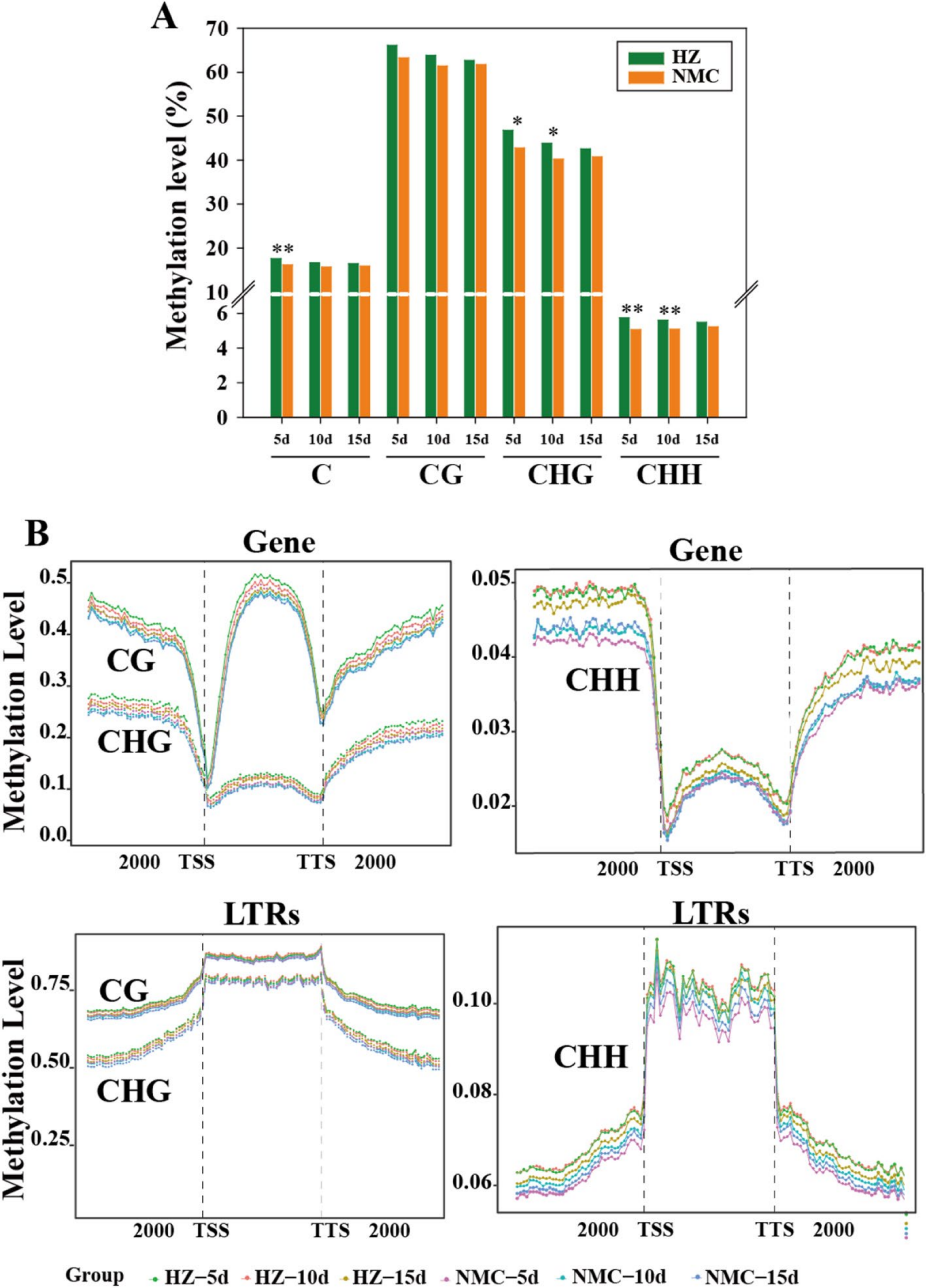
DNA methylation level in ‘HZ’ seeds was higher than those in ‘NMC’ seeds during the early stages of seed development. **A** DNA methylation levels (%) of CG, CHG, and CHH sequence contexts are represented respectively as vertical bar charts. Asterisks indicate a significant difference (t-test: * P < 0.05, ** P < 0.01). **B** DNA methylation levels in the gene body, upstream and downstream (2 kb) regions of genes, and long terminal repeats (LTRs) transposable elements in different sequence contexts (CG, CHG, and CHH) at 5 DAP, 10 DAP and 15 DAP in ‘HZ’ and ‘NMC’

To examine the differences in DNA methylation patterns between the seeds of 'HZ' and 'NMC', we firstly compared the average DNA methylation levels in C contexts between these two cultivars. As depicted in Fig. 3A, the average DNA methylation levels in C contexts at 5 DAP, 10 DAP, and 15 DAP were higher in 'HZ' compared to 'NMC'.

Secondly, we observed that the average DNA methylation levels in CHH, CHG, and CG contexts were all higher in 'HZ' compared to 'NMC'. We conducted a *t*-test to perform statistical analysis and the results revealed that the methylation level in the CHG context was significantly higher in 'HZ' compared to 'NMC' at 5 DAP and 10 DAP, and the methylation level in the CHH context was extremely significantly higher in 'HZ' compared to 'NMC' at 5 DAP and 10 DAP. However, no significant difference in CG methylation levels was observed between 'HZ' and 'NMC' (Fig. 3A; Table S2). Moreover, we discovered that the levels of CHH methylation were higher on 15 chromosomes in 'HZ' than that in 'NMC' (Fig. 2E-F, S2).

Lastly, we computed the DNA methylation levels in the gene body, upstream, and downstream (2 kb) regions of genes, as well as in long terminal repeats (LTRs) of transposable elements. We observed that during the early stage of seed development, the levels of DNA methylation in the CHH context of genes and LTRs, as well as in the CHG context of the gene body, were significantly higher in 'HZ' than in 'NMC' (Fig. 3B).

Collectively, these findings indicate that the disparity in DNA methylation in seeds at the early developmental stage between 'HZ' and 'NMC' is primarily contributed by methylation in the CHH and CHG context.

### Differentially methylated regions and differentially expressed genes are enriched in the ROS pathway

**Fig. 4:**
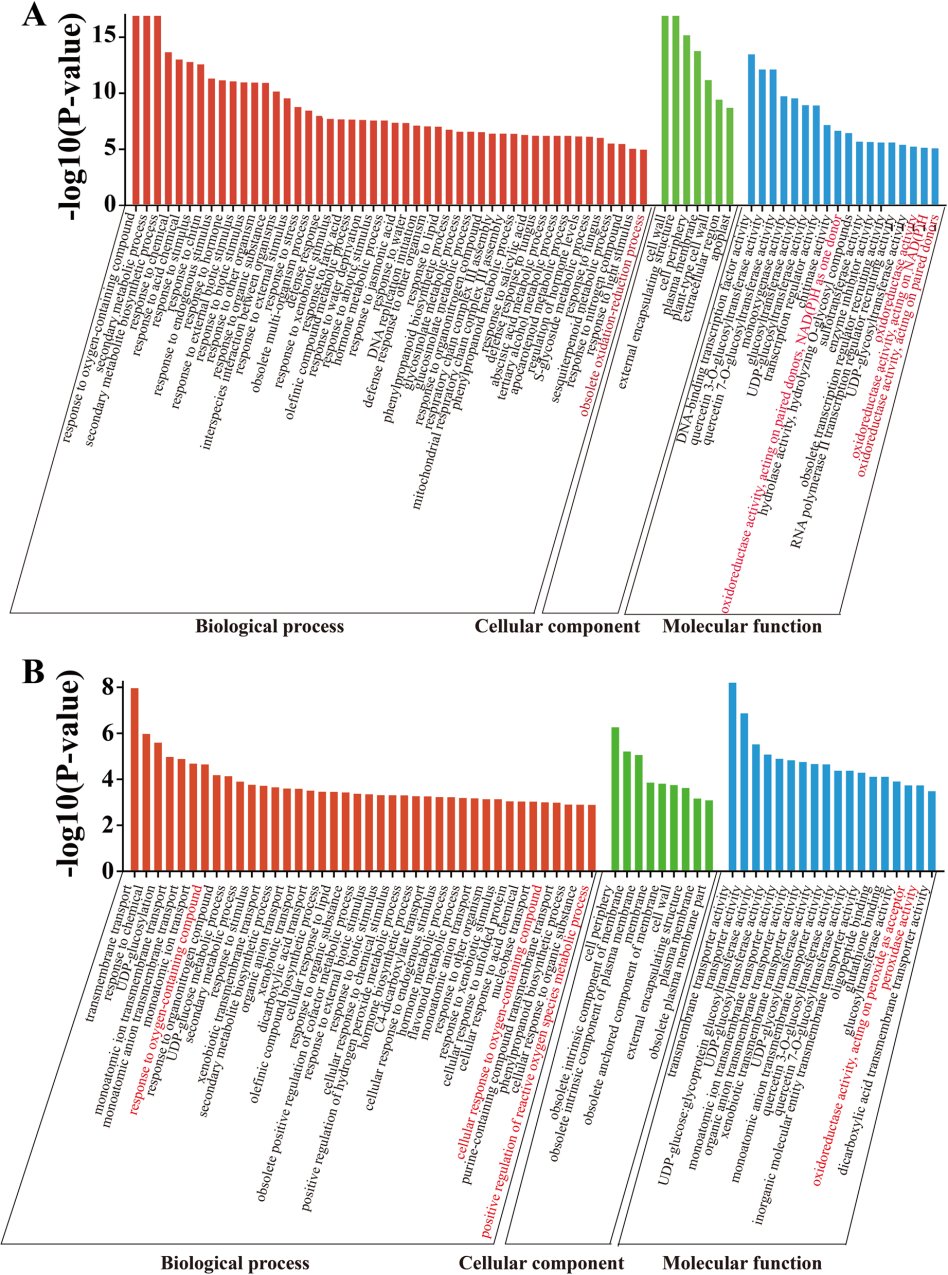
GO term enrichment analysis of genes in DMRs or DEGs. **A** GO term enrichment analysis of the differentially expressed genes (DEGs). **B** GO term enrichment analysis of genes in differentially methylated regions (DMRs) in CHH. Words with the red color indicate the biological process associated with the oxidation reaction pathway

To elucidate the underlying DNA methylation-mediated mechanisms responsible for seed abortion in litchi, we conducted GO term enrichment analysis utilizing differentially expressed genes (DEGs) [Fold change ≥ 2 or ≤ -2] and differentially methylated regions (DMRs) between 'HZ' and 'NMC' seeds. The results showed that a biological process associated with the oxidation reaction pathway was shared among the enriched processes of these DEGs and DMRs in CG, CHG, and CHH contexts (Fig. 4, S3; Table S3), suggesting the existence of differential intracellular oxidative levels during the early developmental stages of seeds in 'HZ' and 'NMC'.

**Fig. 5:**
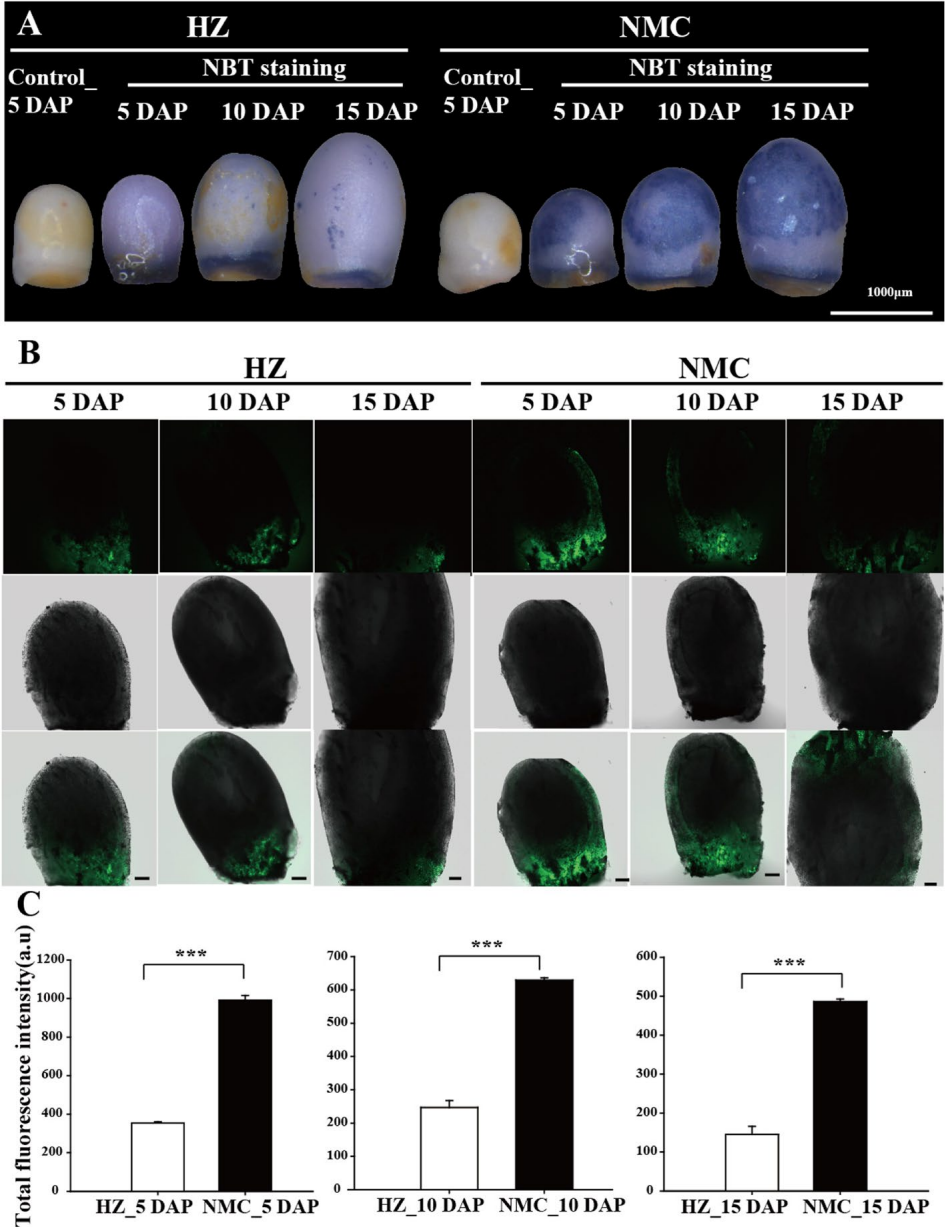
Comparable assays of reactive oxygen species production in the seeds between ‘HZ’ and ‘NMC’. **A** Nitroblue tetrazolium (NBT) was used to detect the accumulation of superoxide and hydrogen peroxide (H_2_O_2_) in the ‘HZ’ and ‘NMC’ seeds at 5 DAP, 10 DAP, and 15 DAP. Scale bars are 1000 μm; **B** Dichlorodihydrofluorescein (DCF) fluorescence of ROS in longitudinal sections of the seeds between 'HZ' and 'NMC'. Scale bars are 100 μm. **C** Quantification of the DCF fluorescence intensity in the seeds of 'HZ' and 'NMC’. At least three independent experiments with three samples each were performed. Asterisks indicate a significant difference (Independent-Sample *t*-test: *** P < 0.001)

To test the hypothesis mentioned above, we performed nitroblue tetrazolium (NBT) staining to assess the levels of superoxide and H_2_O_2_ in the 'HZ' and 'NMC' seeds. As depicted in Fig. 5A, there was a noticeable accumulation of superoxide and H_2_O_2_ in the 'NMC' seeds at 5, 10, and 15 DAP compared to the 'HZ' seeds. Additionally, we employed 2,7-Dichlorodihydrofluorescein diacetate (DCFH-DA) staining to visualize the production of ROS in the seeds. DCFH-DA is a non-fluorescent dye in its reduced form, which is converted into a fluorescent form upon oxidation by H_2_O_2_, hydroxyl radicals, or other by-products of H_2_O_2_ (Sakamoto et al. 2008). Consistent with the NBT staining results, the longitudinal sections of 'NMC' seeds exhibited significantly higher DCF fluorescence signals compared to the 'HZ' seeds at 5, 10, and 15 DAP (Fig. 5 B-C). These findings indicate that 'NMC' seeds generate higher levels of ROS than 'HZ' seeds during the early developmental stages.

**Fig. 6:**
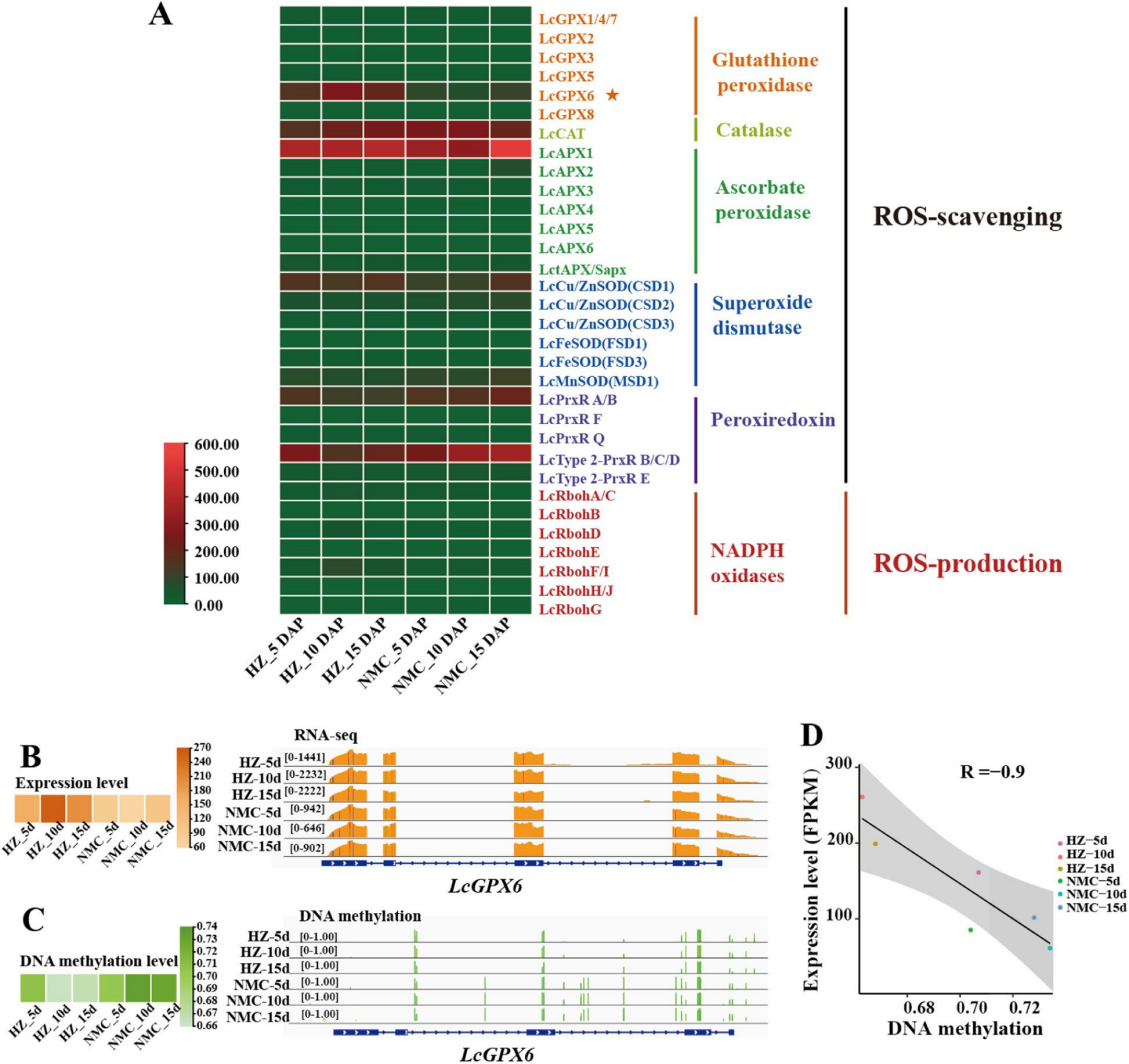
Expression patterns of *LcGPX6* in ‘HZ’ and ‘NMC’ seeds. **A** A heat map showing the expression patterns of genes involved in the production and scavenging of ROS in the seeds of ‘HZ’ and ‘NMC’. **B** A heat map (left panel) and genome browser view (wiggle plots) (right panel) indicate that the expression level of *LcGPX6* was higher in ‘HZ’ seeds than that in ‘NMC’ seeds at 5 DAP, 10 DAP, and 15 DAP. **C** A heat map (left panel) and genome browser view (vertical lines) (right panel) indicate that the DNA methylation level of *LcGPX6* was lower in ‘HZ’ seeds than that in ‘NMC’ seeds at 5 DAP, 10 DAP, and 15 DAP. **D** The correlation analysis of expression level and DNA methylation level of *LcGPX6*

To investigate the underlying reasons for the higher ROS levels in 'NMC' seeds during early developmental stages, we analyzed the expression levels of genes involved in ROS production and scavenging. A total of 32 genes in the litchi genome were identified, including 6 *LcGPXs*, 1 *LcCAT*, 7 *LcAPXs*, 6 *LcSODs*, 5 *LcPrxRs*, and 7 *LcRbohs*. Remarkably, we observed a significant reduction in the expression level of *LcGPX6* in 'NMC' seeds compared to 'HZ' seeds at 5, 10, and 15 DAP (Fig. 6A-B). And a substantial increase in CG methylation level in the gene body of *LcGPX6* was detected in 'NMC' seeds compared to 'HZ' seeds by analyzing the genomic data (Fig. 6C). Additionally, we also found a significant negative correlation between the methylation level and expression level of *LcGPX6* during the early seed development (R = -0.9) (Fig. 6D).

Collectively, these findings suggest that the lower expression level of *LcGPX6* in 'NMC' seeds during early developmental stages could be attributed to the enhanced DNA methylation.

### Ectopic expression of *LcGPX6* in *Arabidopsis* causes defects in seed development

**Fig. 7:**
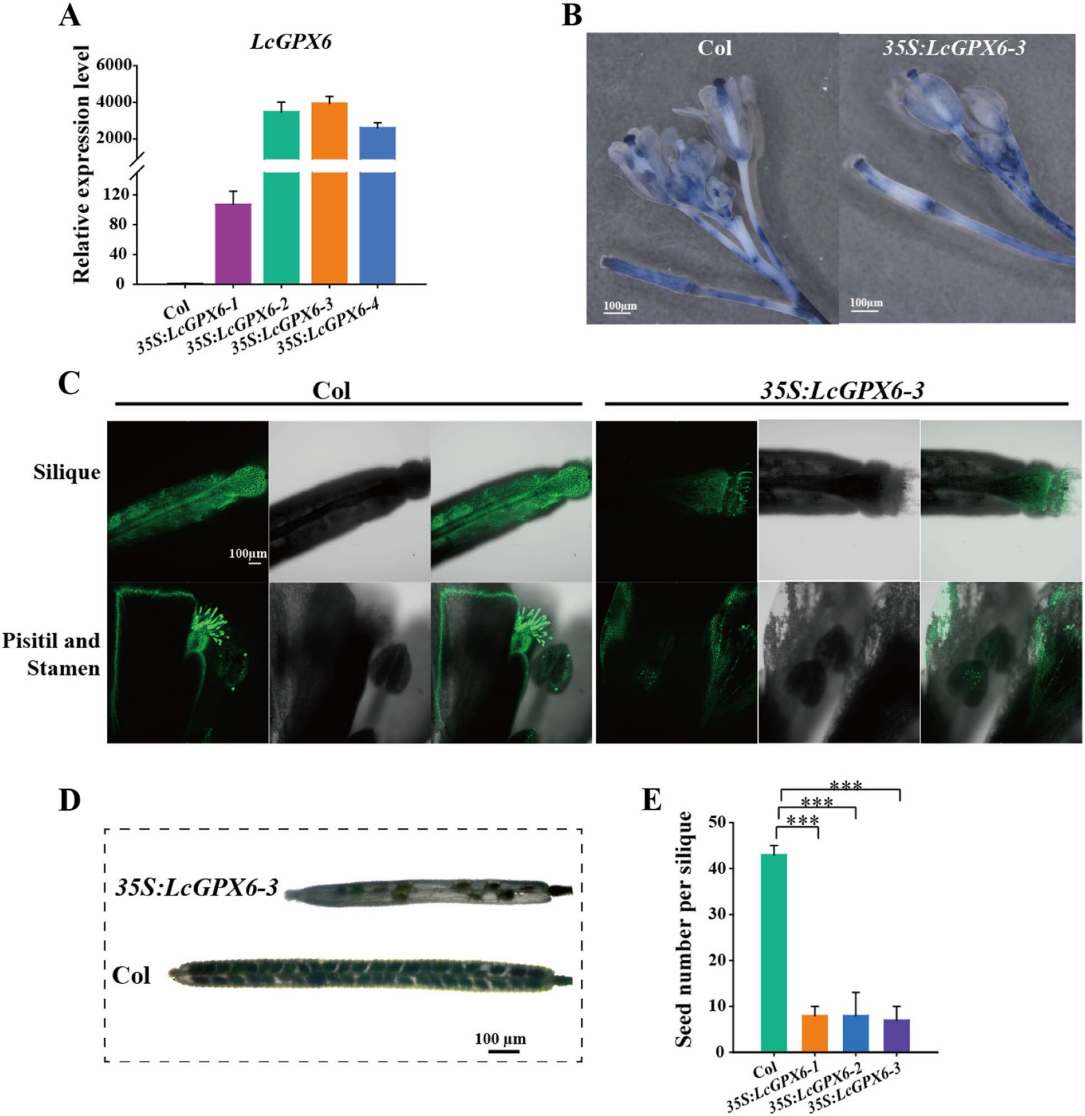
Ectopic expression of *LcGPX6* in *Arabidopsis* causes defects in seed development. **A** Ectopic expression levels of *LcGPX6* in wild type Col and different transgenic lines. qRT-PCR was performed and the *Ubiquitin 10* from *Arabidopsis* was used as the internal reference to analyze the relative level of gene expression. **B** NBT was used to detect the superoxide and H_2_O_2_ accumulation in the inflorescences of Col and *35S:LcGPX6-3* transgenic plants. Scale bars are 1000 μm. **C** DCF fluorescence images of silique, pisitil and stamen between the Col and *35S:LcGPX6-3* transgenic plants. Scale bars are 100 μm. **D** Phenotype of the siliques and seeds in wild type Col and *35S:LcGPX6-3* transgenic plants. Scale bars are 100 μm. **E** Quantitative assay of seed number per silique in wild type Col and *LcGPX6* transgenic lines. Error bar indicates mean ± SD (n = 10 siliques). Statistical significance was determined via employing Independent-Sample *t*-test (***P < 0.001)

To investigate the potential contribution of LcGPX6 in seed development, we conducted ectopic expression experiments in *Arabidopsis*. Four transgenic lines, which displayed relatively higher expression levels of *LcGPX6*, were selected for further analysis (Fig. 7A). Notably, the *LcGPX6* transgenic plants displayed abnormal growth and development, with thin, elongated, and twisted leaves compared to the control. The inflorescence also failed to stand upright and became bent. In addition, siliques were observed to grow from axillary buds on the stems (Figure S4).

To investigate whether the ectopic expression of *LcGPX6* affected ROS levels, NBT and DCFH-DA staining were used. NBT staining revealed a significant reduction in superoxide and H_2_O_2_ accumulation in the siliques, inflorescences, and leaves of *35S:LcGPX6-3* transgenic plants compared to the Col (Figs. 7B, S4). Similarly, DCFH-DA staining showed significantly lower DCF fluorescence signals in the siliques, inflorescences, and leaves of *35S:LcGPX6-3* transgenic plants compared to the Col (Figs. 7C, S4). More importantly, we observed that the silique length and seed number per silique were significantly reduced in *35S:LcGPX6-3* transgenic plants compared to the Col. The *35S:LcGPX6* transgenic lines had approximately 8 seeds per silique, while the wild type Col plants exhibited normal silique development and produced 43 seeds per silique (Fig. 7 D-E). These findings collectively indicate that LcGPX6 plays a significant role in scavenging ROS in *Arabidopsis*, thereby affecting seed development.

## Discussion

Seed development is a tightly regulated process in plants, involving various genetic and epigenetic mechanisms (Han et al. 2019; Verma et al. 2022)). DNA methylation, an epigenetic modification, has been shown to play a crucial role in seed development, however, the underlying mechanism are far from understood. Here, we report the role and regulatory mechanisms of DNA methylation-mediated ROS production in litchi seed abortion.

Normal seed development in crops such as rice and chickpea are known to rely on high levels of DNA methylation (Zhang et al. 2018; Rajkumar et al. 2020). Similarly, our investigation revealed that during the early stages of litchi seed development, the DNA methylation levels in the CHH and CHG sequence contexts were significantly higher in the large-seeded cultivar 'HZ' compared to the seed-abortive cultivar 'NMC' (Fig. 3A). We observed notable differences in CHH context methylation levels in the body, upstream, and downstream regions (2 kb) of genes and LTRs, and CHG context methylation levels in the gene body between 'HZ' and 'NMC' at 5, 10, and 15 DAP (Fig. 3B). However, the relationship between DNA methylation and gene transcription was found to be similar in both 'HZ' and 'NMC'. Notably, the impact of DNA methylation levels in the CG, CHG, and CHH sequence contexts within the gene body and the surrounding region (± 2 kb) on gene expression varied. Our findings demonstrate that DNA methylation can both suppress gene transcription and activate gene expression (Fig. 3). Moreover, the distribution patterns and relationships among transposon density, gene density, CG context methylation, CHG context methylation, CHH context methylation, and gene expression level across the chromosomes were consistent between 'HZ' and 'NMC', with the main difference being the levels of DNA methylation in the CHH and CHG sequence context (Fig. 2E-F). Studies in *Arabidopsis* have shown that genome-wide hypomethylation in the CG sequence context leads to abnormal seed development (Xiao et al. 2006; Mathieu et al. 2007), while loss of non-CG methylation has no effect on seed development (FitzGerald et al. 2008; Lin et al. 2017). However, loss of non-CG methylation has been found to adversely affect ovule development in maize (Garcia-Aguilar et al. 2010). Additionally, maintaining DNA methylation levels in the CHH sequence context through the RdDM-mediated pathway is essential for normal seed development in chickpea (Rajkumar et al. 2020). Based on our study, we propose that the genome-wide hypomethylation levels observed in 'NMC', particularly in the CHH and CHG sequence contexts, are associated with seed abortion.

Although the function of DNA methylation in regulating seed development has been elucidated, the underlying mechanisms remain poorly understood. In order to identify the key biological pathways responsible for the differences in seed development between the 'HZ' and 'NMZ' cultivars, we performed GO enrichment analysis of differentially methylated regions (DMRs) genes or differentially expressed genes (DEGs) (Figs. 4, S3; Table S3). Interestingly, we observed that the enriched biological processes of both DMR and DEG genes contained a pathway related to ROS metabolism. It is widely recognized that the maintenance of intracellular ROS homeostasis is crucial for normal plant growth and development, as excessive ROS production can be cytotoxic and lead to oxidative damage. For instance, in *Arabidopsis*, it has been suggested that maintaining ROS homeostasis is essential for proper embryo development, as high levels of ROS accumulation in embryo sacs can result in sterility or arrested embryogenesis (Victoria Martin et al. 2013). In our study, supporting the findings that genes enriched in the ROS pathway exhibited differential expression between the 'HZ' and 'NMC' cultivars, we found that ROS accumulation in the early developing seeds was significantly higher in the abortive-seeded cultivar 'NMC' compared to the large-seeded cultivar 'HZ', as evidenced by NBT and DCFH-DA staining (Fig. 5). Therefore, we propose that DNA methylation-mediated excessive ROS accumulation in 'NMC' disrupts intracellular ROS homeostasis during early embryo development, ultimately leading to seed abortion. Recent reports have highlighted the critical roles of DNA methylation-mediated ROS homeostasis in various aspects of plant development and stress response, including salt stress (Chen et al. 2015; Hu et al. 2021), heat stress (Ma et al. 2018; Sakai et al. 2022; Zhu et al. 2021), chilling/freezing stress (Guo et al. 2019; Zheng et al. 2022), and fruit ripening (He et al. 2020). Based on our findings, we suggest that DNA methylation-mediated ROS homeostasis also plays a role in seed development in litchi.

The maintenance of ROS homeostasis relies on several key enzymes (Mittler et al. 2004). Among them, glutathione peroxidases (GPXs) play a crucial role in protecting cells from oxidative damage by catalyzing the reduction of H_2_O_2_ or organic hydroperoxides (Pitsch et al. 2010). The observed differences in ROS accumulation levels between the 'NMC' and 'HZ' seeds suggest a disparity in ROS metabolism during the seed development (Fig. 5). As a result, we found that the expression level of *LcGPX6* was significantly lower in 'NMC' seeds compared to 'HZ' seeds during the early developmental stage (Fig. 6). This suggests that the seed abortion in 'NMC' is likely due to the low expression of *LcGPX6*, which leads to the accumulation of excess ROS. Furthermore, when *LcGPX6* was ectopically expressed in *Arabidopsis*, the *LcGPX6* transgenic plants exhibited an enhanced ability to scavenge ROS production and showed significantly lower accumulation of ROS, thereby reducing the seed number per silique (Figs. 7, S4). These findings provide strong evidence for the involvement of LcGPX6 in regulating ROS homeostasis during seed development. It is of note that we have also demonstrated that the accumulation of excess ROS is harmful to seed development in litchi. Conversely, the reduced level of ROS has also been shown to contribute to abnormal seed development in *Arabidopsis*. These findings indicate that maintaining ROS homeostasis is crucial for the normal development of seeds in plants.

**Fig. 8:**
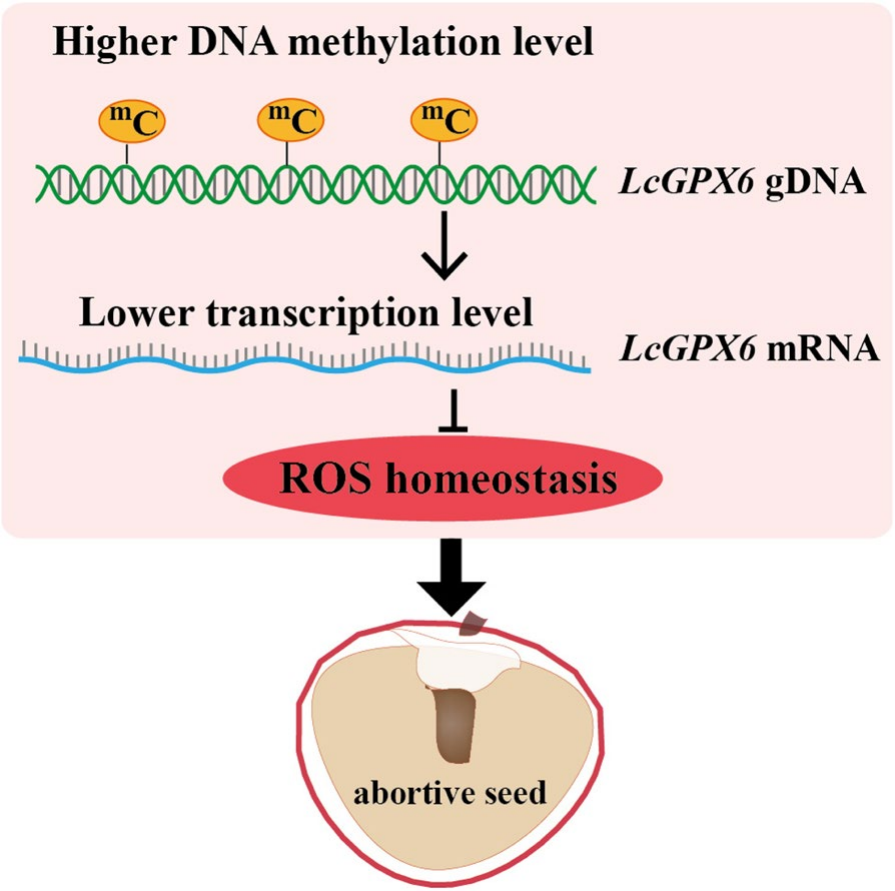
Proposed model of DNA methylation-mediated ROS production contributes to seed abortion in litchi. During the seed development of seed-abortive cultivars in litchi like 'NMC', it was observed that the genomic DNA of *LcGPX6* exhibited relatively higher levels of DNA methylation. This higher methylation resulted in a lower expression level of *LcGPX6*. Since *LcGPX6* is a key gene that encodes an enzyme responsible for scavenging reactive oxygen species (ROS), the decreased expression level of *LcGPX6* may lead to an imbalance in ROS homeostasis, therefor resulting in the production of abortive seeds within the fruit

Although the involvement of DNA methylation-mediated regulation of ROS homeostasis in various aspects of plant development and stress response has been reported, the underlying mechanisms remain largely unclear. In a cotton study, it was observed that RBOH genes were upregulated under heat treatment, accompanied by promoter region hypomethylation and increased H_2_O_2_ concentrations, suggesting that hypomethylation could enhance the expression of RBOH genes, leading to elevated H_2_O_2_ production under heat stress (Ma et al. 2018). In our investigation, we observed hypermethylation in the gene body of *LcGPX6* in 'NMC' seeds compared to 'HZ' seeds during the early developmental stage (Fig. 6). Based on these findings, we propose that the relatively higher ROS production in 'NMC' seeds may be associated with the hypermethylation-mediated downregulation of *LcGPX6* expression, resulting in seed abortion during the early developmental stage (Fig. 8). Further in vivo experiments are necessary to validate the role of LcGPX6 in regulating seed development in litchi.

## Materials and methods

### Plant materials and treatment

The plant materials used in this study were three 20-year-old litchi trees (*Litchi chinensis* Sonn.) of the cultivars 'Huaizhi' ('HZ') and 'Nuomici' ('NMC'). These trees were selected as three biological replicates from the China National Litchi Germplasm Resource Nursery, Institute of Fruit Tree Research, Guangdong Academy of Agricultural Sciences. Seeds at three developmental stages (5 DAP, 10 DAP, and 15 DAP) from 'HZ' and 'NMC' were collected from the China National Litchi Germplasm Resource Nursery, Institute of Fruit Tree Research, Guangdong Academy of Agricultural Sciences, for the purpose of semi-thin sectioning, whole-genome bisulfite sequencing and RNA sequencing.

### Semi-thin sectioning

Seeds of 'HZ' and 'NMC' were collected at 5 days after pollination (DAP). The seeds were carefully separated and pre-fixed overnight at 4 °C in a solution containing 3% paraformaldehyde and 2% glutaraldehyde. After a 30-min rinse in a 0.1 M phosphate buffer (pH 7.4), the seeds were fixed in osmic acid for 4 h. Subsequently, the seeds were rinsed with double distilled water for 30 min, repeating this process four times. The seeds were then immersed in uranyl acetate overnight at 4 °C for block staining. Following another round of rinsing with double distilled water for 20 min, repeating four times, the seeds underwent a dehydration process using increasing concentrations of ethanol. The seeds were then infiltrated with acetone and Epon 812 epoxy resin (Sigma 45,359). Finally, the material was embedded in Epon 812 epoxy resin overnight at 65 °C. For observation, 2 μm sections were cut using a Leica RM2235 microtome with a glass knife and stained with toluidine blue. The sections were examined and photographed using a light microscope.

### Whole-genome bisulfite sequencing

The genomic DNA was extracted using the CTAB Plant Genome DNA Rapid Extraction Kit (Aidlab Biotechnologies). Subsequently, 2 μg of the purified DNA was fragmented and purified. The fragments were then subjected to bisulfite modification using the Methylation-Gold kit (ZYMO, CA, USA). Gene Denovo Biotechnology Co. (Guangzhou, China) performed sequencing using the Illimina HiseqTM 2500 platform. The resulting clean reads were aligned to the litchi genome using BSMAP (Li and Li, 2009). To ensure high quality, reads containing more than 10% unknown nucleotides (N) and 40% low-quality bases (Q-value ≤ 20) were removed. Methylation levels were calculated by determining the percentage of cytosine methylation across the genome, in each chromosome, and in different genomic regions in the context of CG, CHG, and CHH sequences. To identify differentially methylated regions (DMRs) between two samples, a minimum read coverage of 4 was used to determine the methylation status of a base. WGBS was performed on two biological replicates for each stage of seed development. The sequencing depth of the samples ranged from 19 to 23 X (Table S4) and the C-to-T conversion rate was evaluated for all samples (Table S5).

### RNA-seq analysis

Total RNA was extracted from seeds using Trizol (Invitrogen, Carlsbad, CA, USA). Gene Denovo Biotechnology Co. performed sequencing using the Illumina Novaseq6000 platform (Guangzhou, China). To obtain high-quality clean reads, sequences containing adapters, more than 10% unknown nucleotides (N), or 50% low-quality bases (Q-value ≤ 20) were filtered using fastp (version 0.18.0) (Chen et al. 2018). The filtered reads were then aligned to the litchi genome using HISAT2 (Kim et al. 2015). The expression level of each transcription region was quantified using the FPKM (fragment per kilobase of transcript per million mapped reads) value, calculated with RSEM software (Li and Dewey 2011). Differentially expressed genes (DEGs) between two groups were determined using DESeq2 software (Love et al. 2014). Genes/transcripts with a false discovery rate (FDR) < 0.05 and an absolute fold change ≥ 2 were considered as DEGs. Gene Ontology (GO) enrichment analysis was performed using TBtools software (Chen et al. 2020).

### Detection of H_2_O_2_ levels

The litchi seeds/*Arabidopsis* plants were subjected to vacuum treatment for a duration of 2 h/30 min, respectively. Subsequently, the litchi seeds/*Arabidopsis* plants were incubated in a 0.1% NBT solution at room temperature for a period of 20 h/2 h to visualize endogenous H_2_O_2_ and O_2_^−^. During this incubation, the samples were subjected to slow vibration. Following the incubation, the samples were immersed in 95% ethanol that had been heated by boiling water for 20 min to eliminate the green background. For each staining, a total of ten litchi seeds and three *Arabidopsis* plants were utilized and images were captured using a stereomicroscope (ZEISS). To detect intracellular H_2_O_2_, the litchi seeds and *Arabidopsis* plants were incubated in a solution of 100 μM DCFH-DA at a temperature of 37 °C for 1 h [32]. Concurrently, the samples were subjected to slow vibration. The samples were then washed three times with distilled water to remove any residue. The fluorescence of DCF was visualized using confocal laser scanning microscopy (LSM) with excitation at 488 nm (ZEISS LCM-800). Image J software was employed to quantify the intensity of fluorescence (Yang et al. 2018).

### Analysis and treatment of *LcGPX6* transgenic *Arabidopsis*

The coding sequence of *LcGPX6* was fused into the vector pCAMBIA1302 followed by transformation of contructs into *A. tumefaciens*. Later on, *LcGPX6* was introduced into *Arabidopsis* using the floral dip technique (Clough and Bent 1998). For seed development analysis, T3 homozygous plants were employed. A quantitative assay was conducted to determine the seed number per silique in the wild type Col and *LcGPX6* transgenic lines. Statistical significance was determined via employing Independent-Sample *t*-test (***P < 0.001). The primers for generation of *LcGPX6* transgenic *Arabidopsis* are listed in Table S6.

### Supplementary Information

**Additional file 1: Figure S1.** The relationship between DNA methylation and gene transcription in 'HZ' and 'NMC’. **Figure S2.** Chromosome heat maps depicting gene density, transposable elements density, and DNA methylation. **Figure S3.** GO term enrichment analysis of genes in DMRs. **Figure S4.** Ectopic expression of *LcGPX6* in *Arabidopsis* affects plant development.

**Additional file 2: Table S1.** The number and proportion of mCG, mCHG, mCHH in all mC. **Table S2.** DNA methylation levels in C, CG, CHG and CHH sequence context. **Table S3.** The DNA methylayion level of genes in DMRs. **Table S4.** Mapped ratio (%) and sequencing depth of all samples. **Table S5.** Genome coverage of all samples. **Table S6.** Primers used in this study.

## Data Availability

The data underlying this article are available in the article and in its online supplementary material.

## Abbreviations

APX: Ascorbate peroxidase
CAT: Catalase
CMT2: Chromomethylase 2
CMT3: Chromomethylase 3
DAP: Days after pollination
DEGs: Differentially expressed genes
DMRs: Differentially methylated regions
DRM2: Domains Rearranged DNA Methyltransferases 2
GPX: Glutathione peroxidase
H_2_O_2_: Hydrogen peroxide
LTRs: Long Terminal Repeats
mCs: Methylcytosines
MET1: DNA Methyltransferase 1
NBT: Nitroblue tetrazolium
O_2_: Superoxide anion
PrxR: Peroxiredoxin
RdDM: RNA-directed DNA methylation
ROS: Reactive oxygen species
SOD: Superoxide dismutase
TE: Transposable element
TSS: Transcription starting site
TTS: Transcription terminal site
WGBS: Whole Genome Bisulfite Sequence
