# Correction: DNA methylation-mediated ROS production contributes to seed abortion in litchi

**DOI:** 10.1186/s43897-024-00099-y

**Published:** 2024-05-27

**Authors:** Hanhan Xie, Yedan Zheng, Mengyue Xue, Yulian Huang, Dawei Qian, Minglei Zhao, Jianguo Li

**Affiliations:** State Key Laboratory for Conservation and Utilization of Subtropical Agro-Bioresources, Guangdong Laboratory for Lingnan Modern Agriculture, Key Laboratory of Biology and Genetic Improvement of Horticultural Crops (South China), Ministry of Agriculture and Rural Affairs, Guangdong Litchi Engineering Research Center, College of Horticulture, South China Agricultural University, Guangzhou, 510642 China

**Correction** **:** **Mol Horticulture 4, 12 (2024)**

**https://doi.org/10.1186/s43897-024-00089-0**

After publication of this article (Xie et al. 2024), it was brought to our attention that the authors affiliations need to be corrected:

The incorrect author list and affiliations:

Hanhan Xie1,3, Yedan Zheng1,3, Mengyue Xue1,3, Yulian Huang1,3, Dawei Qian1,3, Minglei Zhao1,2,3,4* and Jianguo Li1,2,3,4*

1 Guangdong Laboratory for Lingnan Modern Agriculture, Guangzhou, 510642, China

2 State Key Laboratory for Conservation and Utilization of Subtropical Agro-Bioresources, South China Agricultural University, Guangzhou, 510642, China

3Key Laboratory of Biology and Genetic Improvement of Horticultural Crops (South China), Ministry of Agriculture and Rural Affairs, College of Horticulture, South China Agricultural University, Guangzhou, 510642, China

4Guangdong Litchi Engineering Research Center, College of Horticulture, South China Agricultural University, Guangzhou, 510642, China

The correct affiliation for all authors:

State Key Laboratory for Conservation and Utilization of Subtropical Agro-Bioresources, Guangdong Laboratory for Lingnan Modern Agriculture, Key Laboratory of Biology and Genetic Improvement of Horticultural Crops (South China), Ministry of Agriculture and Rural Affairs, Guangdong Litchi Engineering Research Center, College of Horticulture, South China Agricultural University, Guangzhou, 510642, China

The original publication has been corrected.
