# Supplementary material for: DNA methylation-mediated ROS production contributes to seed abortion in litchi
Source: Mol Hortic. 2024 Apr 2;4:12. doi: 10.1186/s43897-024-00089-0 (PMC10986121; doi:10.1186/s43897-024-00089-0)
Supplement: Supplementary file 1 — Additional file 1: Figure S1. The relationship between DNA methylation and gene transcription in 'HZ' and 'NMC’. Figure S2. Chromosome heat maps depicting gene density, transposable elements density, and DNA methylation. Figure S3. GO term enrichment analysis of genes in DMRs. Figure S4. Ectopic expression of LcGPX6 in Arabidopsis affects plant development. [file 43897_2024_89_MOESM1_ESM.docx]

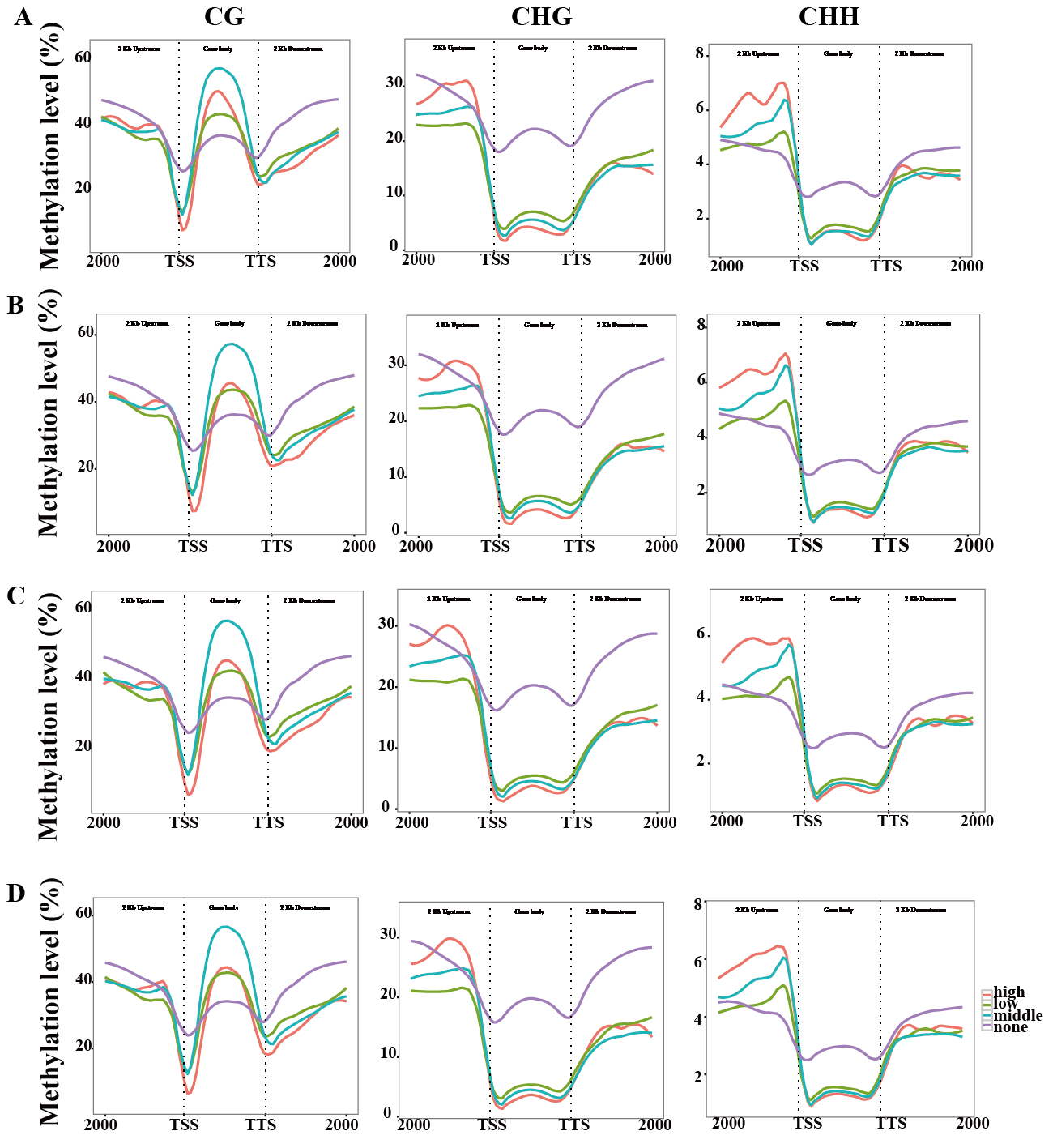


**Figure S1. The relationship between DNA methylation and gene transcription in 'HZ' and 'NMC’.**The DNA methylation levels within the gene body and ±2 kb regions of genes are shown for different sequence contexts in gene sets that are expressed at different levels in ‘HZ’ at 10 DAP (A) and 15 DAP (B). The DNA methylation levels within the gene body and ±2 kb regions of genes are shown for different sequence contexts in gene sets that are expressed at different levels in ‘NMC’ at 10 DAP (C) and 15 DAP (D). The expression levels are categorized as high (FPKM>100), middle (10<FPKM≤100), low (1<FPKM≤10), and none (FPKM≤1). The terms TSS and TTS represent the transcription starting site and transcription terminal site, respectively.


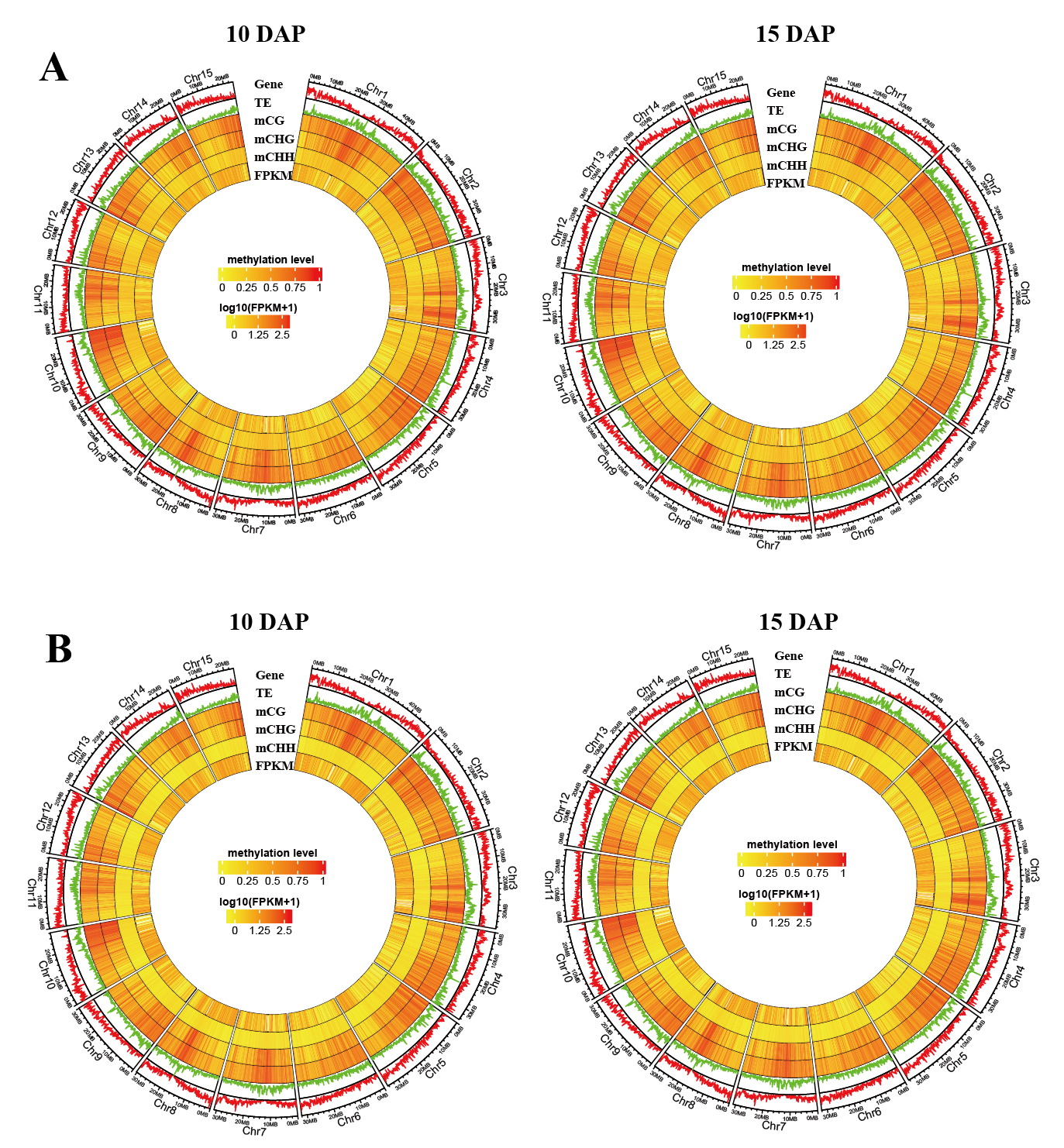


**Figure S2. Chromosome heat maps depicting gene density, transposable elements density, and DNA methylation.**(A) Chromosome heat maps illustrating gene density, transposable elements density, DNA methylation levels in different sequence contexts, and gene expression levels of seeds at 10 DAP and 15 DAP in 'HZ’. (B) Chromosome heat maps illustrating gene density, transposable elements density, DNA methylation levels in different sequence contexts, and gene expression levels of seeds at 10 DAP and 15 DAP in ‘NMC’. The bin size used for this visualization is 100 kb. The outer ring displays the chromosome names and scale.


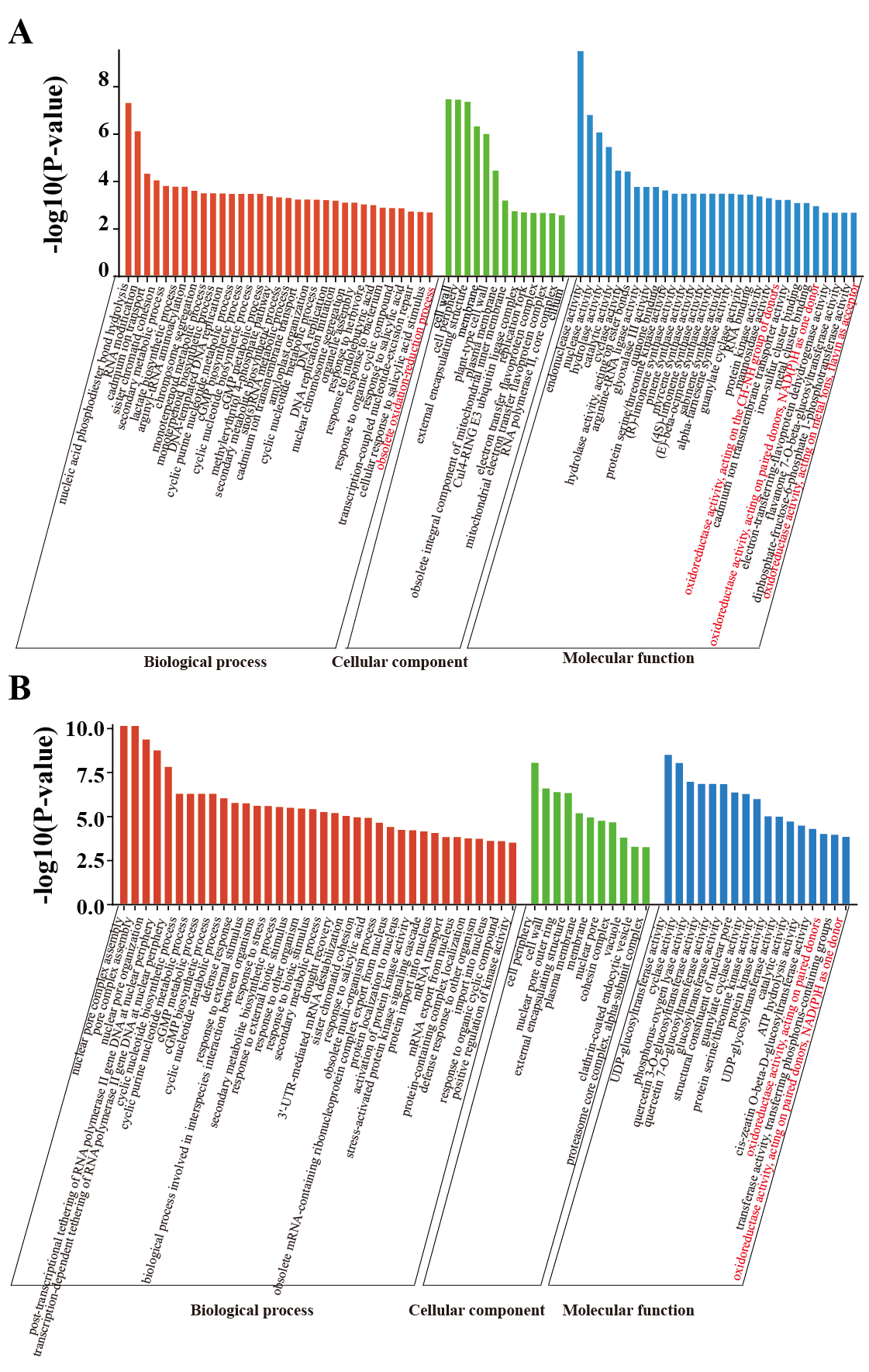


**Figure S3. GO term enrichment analysis of genes in DMRs.**
(A) GO term enrichment analysis of genes in DMRs in CG context. (B) GO term enrichment analysis of genes in DMRs in CHG context. Words with the red color indicate the biological process associated with the oxidation reaction pathway.

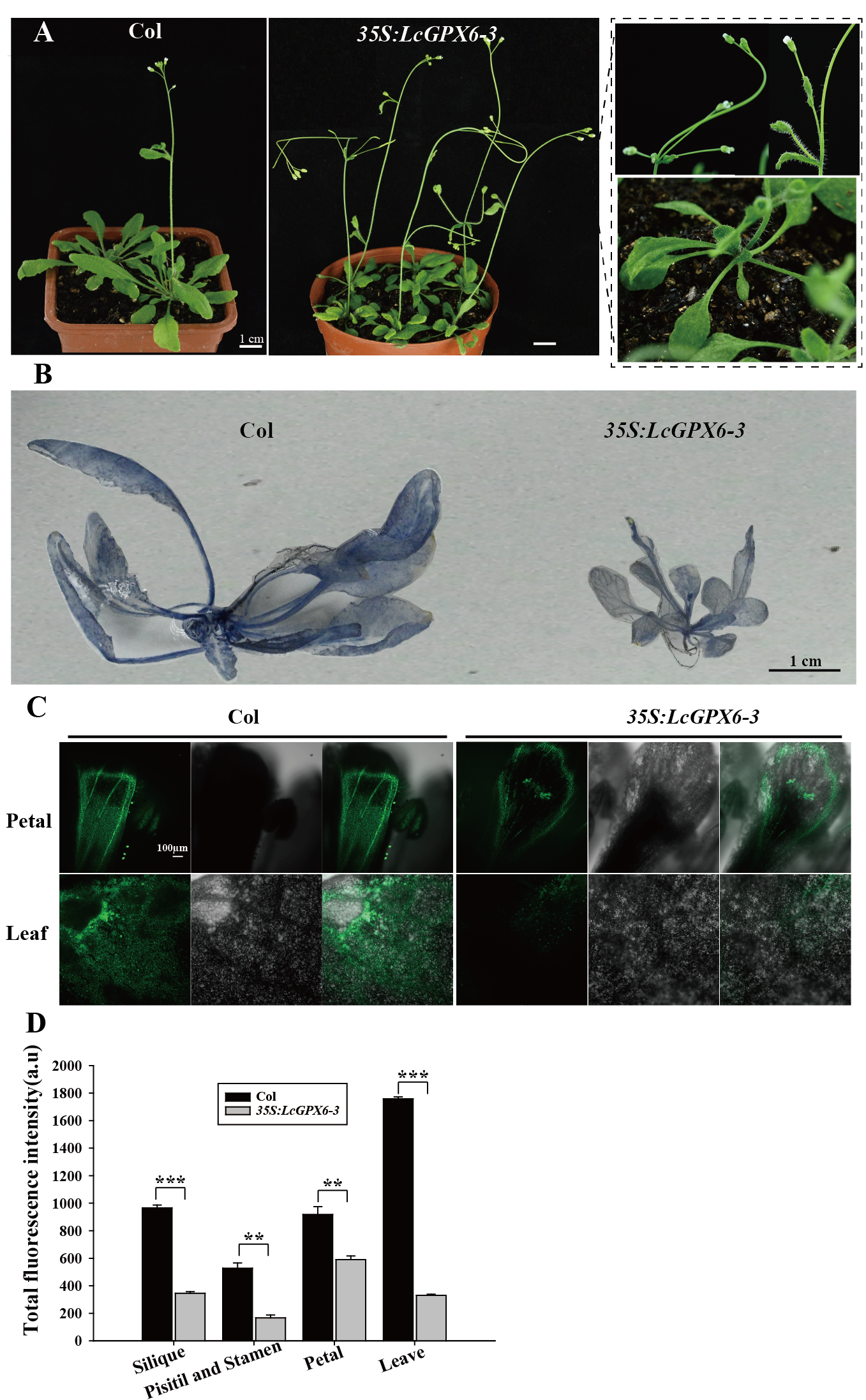
**Figure S4. Ectopic expression of *LcGPX6* in *Arabidopsis* affects plant development.**
(A) Phenotype of leaves and inflorescences in wild type Col and *LcGPX6* transgenic lines. (B) Superoxide and H_2_O_2_ accumulation detected with NBT in leaves between Col and *35S:LcGPX6-3* transgenic plants. Scale bars are 1000 μm. (C) DCF fluorescence images of petals and leaves between Col and *35S:LcGPX6-3* transgenic plants. Scale bars are 100 μm. (D) Total DCF fluorescence quantification of seed, petals and leaves in Col and *35S:LcGPX6* transgenic plants using Image J. At least three independent experiments with three samples each were performed. Asterisks indicate a significant difference (Independent-Sample *t*-test: **P<0.05, ***P < 0.001).
